# Supplementary material for: Acceptance and compliance with micronutrient powder (MNP) among children aged 6–23 months in northern Nigeria
Source: PLOS Glob Public Health. 2022 Oct 17;2(10):e0000961. doi: 10.1371/journal.pgph.0000961 (PMC10022258; doi:10.1371/journal.pgph.0000961)
Supplement: S1 Dataset — (ZIP) [file pgph.0000961.s008.zip › S8_Dataset/In-depth Interviews.zip!/In-depth Interviews/080715_K_J_AB_2_CG.pdf]

I: Good morning ma, thank you for giving me this opportunity to talk to you this morning. I want you to describe for me the people in your immediate family living in your household

R: We are many in this household

I: What about in your family how many children between the ages of 6-23 months

R: These are my children, we are five

I: How old is she

R: 14 months

I: We have heard of food challenges for young children in this setting related to the seasons, like in this raining season what are the types food that are difficult to get like mango (she cuts in)

R: We have mango all the year

I: Okay, like those types of foods that children eat that is not available in this raining season

R: She drink kunu, she eat spaghetti and rice with some other foods

I: Other food like...

R: I have forgotten some of them

I: Like now if there is no rice, kunu or spaghetti what do you give her

R: When we cook tuwo millet, we give her.

I: Thank you very much, there are some women who said they give their children tea, do you know tea

R: Yes I know

I: This tea tell me how do they prepare tea

R: Long silent

I: Do you give your child tea

R: I don't give her tea

I: But do you know how they make tea?

R: Yes in this community I know how they make tea

I: What and what do they put or add inside?

R: Actually, that one I don't know what they put inside, although there was one I once saw, where one particular type of leaves was put in the water with sugar.

I: Which type of water is use to make tea hot or cold water?

R: It is the boiling water

I: Like the last time you were given MNP to give to your child, could you tell me about your experiences with the product?

R: Actually, when I started giving her she started eating well. My friend told me that you brought a medicine that has increase their eating habit without providing us with food to feed them. She eats very well

I: Like what and what does she eat now

R: Like those once I have already mentioned, you know this is a village setting, we give or buy for them what is available like rice or spaghetti where we cannot afford we cook tuwo and to them

I: What about breast milk, has it increase the rate she takes breast milk?

R: Yes she takes breast milk

I: Can you tell me what has made to enjoy giving her this product since you started giving her

R: You should just bring the food we will give to them

I: Before she started taking this product, how her eating habit

R: She was not eating well but now she eats very well

I: What about her health condition

R: Her health condition is good, like yesterday and today she has been suffering from diarrhoea and cough although she has been eating well

I: When you started administering the MNP to her, can you share with me your experiences her?

R: She has been taking the product without any problem because the product is tasteless

I: There are some women that said it was hard to store the MNP because some children will come and steal it, some eaten by rodents. Tell me about your experience

R: Nothing has never happen to mind although there are rodents in our community but my own product is been properly kept

I: You said this product has increase her eating habit do you think it is this product that has cause it

R: Is not this one that has cause it but I thought it is the one

I: Since you started giving her the product have you ever skip any day?

R: Since the very time it was brought to me I have been giving her in the morning and I have not missed a day

I: Some caregivers said they do forget to give their child the MNP, do you have similar problem?

R: Those that do forget may be they have too much work on their head, some again may don't care about the product because even if the mother will forget somebody can just remind her by asking her have you giving your child that medicine from there she will remember

I: If you are to give advice those that do forget what will you say

R: They should test the product and see either the product is effective or not. And if the see that it is not effective they can stop using it

I: This MNP what type of food do you normally use it with

R: As for me, I will always make use of kunu

I: Which type of kunu

R: Millet kunu

I: The millet kunu is in which form

R: The millet kunu is in semi liquid form

I: Is the millet kunu hot or cold that you do used

R: At times it is hot and at times it is cold

I: Thank you very much. Like the first time you started giving the MNP did she noticed any different taste of koko?

R: She didn't noticed anything

I: Thank you very much. In other communities we notice that the sharing of the MNP occurred, could you talk about any sharing of the MNP when you used it with another woman

R: I always fetch the one that will be enough for her

I: You mean you have never experience where her brother will start crying that he too must take and you will give him or take the remaining and give him or anybody

R: I don't usually give her close to them

I: You know you were given 60 sachets for 60 days do you think it will be enough for 60 days?

R: One will be enough

I: Why do you say one will be enough?

R: You know some children are not strong enough to take more than one

I: Thank you very much. Now that you have used the MNP, could you tell me how you perceive it?

R: It is a medicine

I: Why did you say it is a medicine?

R: It is medicine because I have used it and seen its effectiveness

I: Now that you have used it, would you purchase it if it were cheaply on sale in this community?

R: Yes, because if you have use it and see that it is good you can buy and to the person who has not use it will not know of its usefulness and will not buy

I: How much do you think you can but it supposing it is taken to the market?

R: In my own seeing it should not be more than five hundred naira (N500) but to some it can be N200, or N300 but to me once I have use it and see that it is very effective even at N500 I will buy it.

I: Thank you for your rich your information. In this community Hillinde, who are the people that community members respect and obey their instructions?

R: Is the district head, he is the one that can say something and people will accept or say don't do this and nobody will do it.

I: When you first gave birth to your first child, who normally gives you advice

R: Is my mother that taught me how to take care of my child

I: Okay, who taught you of how to administer this MNP to your child?

R: It is the District head

I: Like now if this MNP is brought to the community to get it distributed to the caregivers, where do you think is the right place?

R: At the place of the district head

I: Like now if this MNP is brought to the community, where do you like to hear more information about it?

R: Where the person that got it from should also go there for more information at district head place

I: Why is it that it is only the district head you have been mentioning?

R: He owns the community and equally decides for his people

I: In this community who are the people that caregivers do take and respect their instruction about child feeding

R: It is my husband

I: Apart from the father of the child and the district head, what form of media do you prefer for receiving MNP messages?

R: Let it be through the town crier

I: Where will this town crier come from?

R: The district head will appoint one

I: Can you tell me what made the implementation of tamowa to be successful that you will like us to follow with this MNP

R: (Long silent) I don't know

I: You have been using this product; if you are ask to talk to another female caregiver about this MNP what will you tell her

R: I can tell her what I was told about the product

I: What were you told about the product?

R: That she should use the product with kunu to a little quantity that the child can finished at once, and one sachet should be use per day without missing a day.

I: What will you tell her about the sharing of the product?

R: Only the child should be giving the product without sharing it with another child

I: Now we have come to the end of this interview I don’t know if you have any problem, question about the product since the very day you started using it on your daughter

R: (children making noise), My advice is that since we have accepted what you brought to us and it has increase her eating habit, you should help us and provide us with food that will be use to feed them together with the tamowa

I: Do you have any general question about the size and packaging

R: The size is okay.

I: Thank you very much.
